# Correction to “Menopause, Menstrual Cycle, and Skin Barrier Function”

**DOI:** 10.1111/srt.70274

**Published:** 2025-10-09

Đ. C. Nikoletić, D. Ivanov, O. Levakov, J. Bulajić, S. Lukač, V. K. Rakić, and M. Ivkov‐Simić, “Menopause, Menstrual Cycle, and Skin Barrier Function,” *Skin Research and Technology* 31, no. 7 (2025): e70203, https://doi.org/10.1111/srt.70203.

It has come to the authors’ attention that there was an inadvertent permutation of two terms in the Conclusions section of both the abstract and the main text, as well as an error in Table 2.

Specifically:

- In the “Conclusion” section of the Abstract, the sentence:
  “Our study indicates a more functional epidermal barrier during the ovulatory phase, as evidenced by higher TEWL values and lower SH values compared to the mid‐luteal phase.”
  should read (changes in bold):
  “Our study indicates a more functional epidermal barrier during the ovulatory phase, as evidenced by **lower** TEWL values and **higher** SH values compared to the mid‐luteal phase.”
- In the “Conclusion” section of the main text, the sentence:
  “Our study indicates that the epidermal barrier is more functional during the ovulatory phase, as evidenced by higher TEWL values and lower SH values compared to the mid‐luteal phase.”
  should read (changes in bold):
  “Our study indicates that the epidermal barrier is more functional during the ovulatory phase, as evidenced by **lower** TEWL values and **higher** SH values compared to the mid‐luteal phase.”
- In Table 2, there is an error in the reported SH1 value for participants in the reproductive phase. The value is incorrectly listed as 36.27. The correct value should be 40.55, which is consistent with both Figure 2 and the corresponding description in the Results section.

The authors sincerely apologize for these errors and any confusion they may have caused.

